# Cannabis Use Among Students in Grades 8, 10, and 12, by Sex — King County, Washington, 2008–2021

**DOI:** 10.15585/mmwr.mm7302a1

**Published:** 2024-01-18

**Authors:** Precious Esie, Myduc Ta

**Affiliations:** ^1^Epidemic Intelligence Service, CDC; ^2^Assessment, Policy Development & Evaluation Unit, Public Health — Seattle & King County, Washington.

**Summary**

**What is already known about this topic?**

Cannabis use during adolescence is associated with poor outcomes, including cognitive impairment and impaired driving. Cannabis use among younger persons has been declining, but less is known about sex-specific trends.

**What is added by this report?**

During 2008–2021, in King County, Washington, current cannabis use prevalences among male and female students in grades 8, 10, and 12 declined. During 2008–2014, current-use prevalence was higher among male students than among female students. In 2021, for the first time, current-use prevalence was lower among male students than female students.

**What are the implications for public health practice?**

Developing tailored interventions that consider potential differences in risk and protective factors by sex or gender identity could promote equity in youth cannabis use reduction strategies.

## Abstract

Cannabis use during adolescence is associated with poor outcomes, including cognitive impairment, cannabis use disorder, and impaired driving. To guide prevention and use reduction strategies, Public Health — Seattle & King County described recent trends in cannabis use by sex among King County, Washington students in grades 8, 10, and 12 and examined trends in sex-based differences. Data collected during seven 2008–2021 survey periods by the Healthy Youth Survey (administered by the Washington State Department of Health) and restricted to King County students in grades 8, 10, and 12 (range = 33,439–39,391 students per cycle) were analyzed. Prevalence estimates were generated and sex-based prevalence differences (PDs) in current use (≥1 day during the previous 30 days) and frequent use (≥6 days during the previous 30 days) were assessed. PD models used weighted generalized linear regression with an interaction between sex and survey year. During 2008–2021, cannabis use declined among both male and female students. During 2008–2014, cannabis use was higher among male students than among female students (e.g., PD in 2008 = 4.8%) and not significantly different during 2014–2016; however, in 2021, current-use prevalence was lower among male students than among female students for the first time (PD = −1.3%). Frequent-use prevalence was similar among males and females. By grade levels, the highest prevalence of both current and frequent cannabis use was observed among 12th grade students, followed by 10th and 8th graders. Sex-specific differences by grade mirrored overall patterns. Developing tailored interventions that consider potential differences in risk and protective factors by sex or gender identity could promote equity in youth (grades 8, 10, and 12) cannabis use reduction measures.

## Introduction

Cannabis use during adolescence is associated with poor outcomes, including cognitive impairment, cannabis use disorder (the inability to stop using cannabis despite the presence of health and social problems), and an increased risk for being involved in a motor vehicle collision because of impaired driving (*1*). More frequent use might be a stronger predictor of these outcomes (*1*). In 2012, Washington was among the first states to legalize nonmedical cannabis use for adults aged ≥21 years, prompting concern about how this measure might affect use by younger persons. Multiple factors might lead to increased cannabis use by youths, including increased permissiveness, reduced perception of potential harm, and an increase in alternative consumption methods (e.g., edibles and vaping) (*2*,*3*). Despite these concerns, however, data from Washington suggest that legalization was not associated with increased cannabis use by adolescents and young adults (*4*,*5*). Although the Healthy Youth Survey, administered by Washington State Department of Health, shows overall declines in cannabis use based on data through 2016, less is known about trends among more frequent users, or how trends might have varied by gender or sex assigned at birth (*5*). Historically, prevalence of cannabis use has been higher among male youths than their female counterparts (*6*). However, recent national data indicate a shift, with prevalence now higher among female youths compared with male youths (*7*). To guide prevention and reduction strategies, Public Health — Seattle & King County (PHSKC) described trends and examined sex-based differences in both current and more frequent cannabis use among youths in King County, Washington.

## Methods

### Data Source

The Healthy Youth Survey is a representative, biennial, cross-sectional survey of health and health-related behaviors administered by Washington State Department of Health to public school students in grades 6, 8, 10, and 12. Students complete anonymous self-administered questionnaires during structured classroom time. The Healthy Youth Survey has been conducted in even numbered years, except 2020, when it was delayed until 2021 because of shifts to remote learning during the COVID-19 pandemic (*8*). PHSKC used data from seven survey cycles conducted during 2008–2021, restricting analyses to King County students in grades 8, 10, and 12. The analytic sample from each cycle ranged from 33,439 students in 2021 to 39,391 students in 2016.

### Data Analysis

Current (≥1 day during the previous 30 days) and frequent (≥6 days during the previous 30 days) cannabis use prevalence estimates by sex assigned at birth were generated, and patterns were described. Crude prevalence differences (PDs) and 95% CIs by sex were assessed using separate generalized linear models for each outcome containing a quasibinomial distribution and identity link. Models contained an interaction term between sex and categorical survey year, accounting for variations in PDs over time; PDs corresponded to the coefficients for sex. P-values <0.05 were considered statistically significant, corresponding to estimates for which 95% CIs exclude 0. Lastly, analyses were replicated, stratifying by grade (8, 10, and 12). Analyses used raked weights based on grade by sex by school district margins to be representative of King County public school students in grades 8, 10, and 12. Analyses were conducted using R software (version 4.2.3; R Foundation). This activity was reviewed by CDC, deemed not research, and was conducted consistent with applicable federal law and CDC policy.* 

## Results

During 2008, 2010, and 2012, the prevalence of current cannabis use was stable among both male students (19.2%, 20.4%, and 20.3%, respectively) and female students (14.4%, 14.9%, and 15.5%, respectively) (Table). The 2014 survey cycle identified a decline in current use for male students (from 20.3% in 2012 to 16.4% in 2014), whereas current use remained stable among female students (15.5% in 2012 and 15.2% in 2014). Prevalence of current use was lowest during the 2021 cycle for both male (7.7%) and female students (9.0%). Patterns of frequent cannabis use by sex over time were similar. Likewise, frequent-use prevalence was lowest during the 2021 cycle for both male students (3.7%) and female students (3.6%).

**TABLE:** Weighted* prevalence of current^†^ and frequent^§^ cannabis use among students in grades 8, 10, and 12, by sex assigned at birth — Washington Healthy Youth Survey, King County, Washington, 2008–2021

Survey year	Cannabis use status, % (95% CI)			
	Current use^†^		Frequent use^§^	
	Male	Female	Male	Female
2008	19.2 (17.5–21.1)	14.4 (13.0–16.0)	9.0 (8.0–10.1)	4.7 (4.1–5.4)
2010	20.4 (18.7–22.1)	14.9 (13.7–16.1)	9.8 (8.8–10.9)	5.1 (4.5–5.7)
2012	20.3 (18.5–22.3)	15.5 (14.1–17.0)	10.0 (8.9–11.2)	5.3 (4.7–6.0)
2014	16.4 (14.9–18.0)	15.2 (13.9–16.6)	7.5 (6.6–8.4)	5.4 (4.8–6.0)
2016	15.0 (13.4–16.8)	14.6 (13.1–16.2)	7.2 (6.3–8.2)	5.4 (4.8–6.2)
2018	15.3 (13.9–16.8)	15.1 (13.7–16.6)	6.5 (5.8–7.4)	5.3 (4.7–6.0)
2021	7.7 (6.4–9.3)	9.0 (7.7–10.6)	3.7 (3.0–4.5)	3.6 (3.0–4.4)

* Reflects King County, Washington public school enrollment by grade and sex assigned at birth.

^†^ One or more days during the previous 30 days.

^§^ Six or more days during the previous 30 days.

Examination of current cannabis use by sex revealed that prevalence among male students was significantly higher than that among female students between the 2008 (PD = 4.8%, 95% CI = 3.8%–5.8%) and 2014 (PD = 1.2%, 95% CI = 0.4%–2.0%) cycles (Figure), although sex-specific current-use prevalences among male and female students were not significantly different during the 2016 and 2018 cycles. In 2021, however, current-use prevalence among male students was significantly lower than that among female students, representing a reversal of previous sex-specific differences (PD = −1.3%, 95% CI = −2.1% to −0.5%). Frequent-use prevalence among male students was significantly higher than that among female students across all cycles except 2021, during which no substantial difference existed. During 2021, the highest prevalence of current cannabis use was observed among 12th grade students (males 15.3%, females 17.4%), followed by 10th (males 5.1%, females 7.0%) and 8th graders (males 1.8%, females 2.1%). Similar patterns were seen for frequent cannabis use by grade levels with the highest use among 12th grade students. (Supplementary Table; https://stacks.cdc.gov/view/cdc/140555).

**FIGURE:**
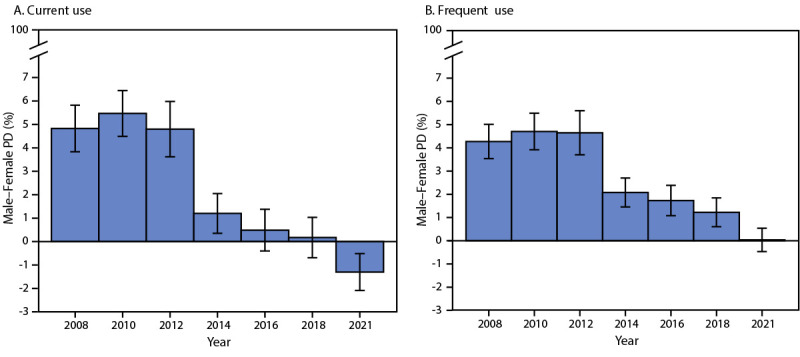
Weighted sex-based prevalence differences* in current^†^ (A) and frequent^§^ (B) cannabis use among students in grades 8, 10, and 12 — Washington Healthy Youth Survey, King County, Washington, 2008–2021^¶ ^ **Abbreviation:** PD = prevalence difference. * With 95% CIs indicated by error bars. ^†^ One or more days during the previous 30 days. ^§^ Six or more days during the previous 30 days. ^¶^ 2020 survey delayed from 2020 until 2021 because of the COVID-19 pandemic.

## Discussion

During 2008–2021, in addition to overall decreases in cannabis use among students in grades 8, 10, and 12 in King County, Washington, a narrowing and possible reversal of sex-based differences in current cannabis use was observed. These reported recent decreases in cannabis use among students in grades 8, 10, and 12 are consistent with overall statewide trends (*8*) and sex-stratified trends in national data showing larger decreases among male students (*7*).

### Decrease in Cannabis Use

The observed overall decreases in cannabis use among students in grades 8, 10, and 12 might be associated with changes in the availability of cannabis among persons aged ≥21 years as well as limited opportunities to engage in use. The period 2012–2014 includes the legalization of nonmedical cannabis in Washington in 2012. Researchers studying the association of cannabis laws with cannabis use among high school students (grades 9–12) have observed similar declines in cannabis use after legalization of nonmedical cannabis (*9*). The legalization of nonmedical cannabis for adults aged ≥21 years in Washington with licensed dispensaries requiring proof of age might have affected availability of cannabis to younger persons as well as their opportunities to engage in its use. This, in turn, might have had an impact on use prevalence. The period 2018–2021 also included the unexpected shift to remote learning environments in 2020 associated with the COVID-19 pandemic. With increased time spent at home, students might have been subject to increased parental supervision, which could deter substance use, including use of cannabis. Increased parental supervision could have been compounded by limited access to cannabis, if a main source was from friends or social settings away from the home.

### Sex Differences in Cannabis Use

Shifts in sex-specific differences in cannabis use raise questions about underlying factors and potential implications for prevention and use reduction strategies for youths. One explanation for diminishing sex-specific differences might be related to a previous focus on higher prevalence users. For example, interventions might have been most effective among males because of their higher use prevalences. A second explanation might be related to evolving social norms regarding cannabis use. Among adolescents, a positive association between cannabis use and norms surrounding its use has been established (*2*). However, whether the strength of the association has changed over time, varies by sex, or has become stronger for females than for males is unclear. Future studies might examine trends in cannabis use norms by sex, and the association between norms and cannabis use by sex.

### Limitations

The findings in this report are subject to at least four limitations. First, because cannabis use was self-reported, use might have been underreported (e.g., because of legal implications). To mitigate this potential bias, the Healthy Youth Survey was administered during structured classroom time in a test-like environment, and no identifying information was collected (*10*). Second, this report relied on self-reported sex assigned at birth to categorize students by sex and does not include students who identify as transgender or nonbinary. The Healthy Youth Survey introduced gender identity questions in 2018; thus, examining trends by gender identity was not possible. Third, this report provides trends in prevalence of cannabis use among King County students in grades 8, 10, and 12, and was not intended to identify contextual factors that might have influenced cannabis use estimates (e.g., legalization or the COVID-19 pandemic). Finally, findings do not necessarily apply to students who are not in grades 8, 10, and 12 enrolled in King County, Washington public schools.

### Implications for Public Health Practice

Although downward trends in cannabis use among King County students in grades 8, 10, and 12 are encouraging, continued monitoring is necessary to better understand longer-term effects of social phenomena, including cannabis legalization and pandemic-related disruptions, and to assess whether observed decreases are sustained. It is important for monitoring to prioritize identifying differences across demographic characteristics, including sex or gender identity, which can potentially support the development of tailored interventions and ensure equity in programmatic cannabis use reduction and prevention measures. Lastly, whereas the focus of the present analysis was on sex, future analyses could explore potential variations across additional demographic variables including race, ethnicity, or socioeconomic status.

## Footnotes

* 45 C.F.R. part 46, 21 C.F.R. part 56; 42 U.S.C. Sect. 241(d); 5 U.S.C. Sect. 552a; 44 U.S.C. Sect. 3501 et seq.
